# Effects of calcium supplementation to resuscitation fluids in endurance horses: A randomized, blinded, clinical trial

**DOI:** 10.1111/jvim.16715

**Published:** 2023-05-02

**Authors:** C. Langdon Fielding, Emma L. Deane, Dustin S. Major, Jennifer R. Mayer, Juliette C. Love, Michael S. Peralez, K. Gary Magdesian

**Affiliations:** ^1^ Loomis Basin Equine Medical Center Penryn California USA; ^2^ Foothill Equine Arcadia California USA; ^3^ The Department of Medicine and Epidemiology, School of Veterinary Medicine University of California Davis California USA

**Keywords:** dehydration, electrolytes, endurance, intravenous

## Abstract

**Background:**

The addition of calcium to resuscitation fluids is a common practice in horses, but studies evaluating the effects of calcium supplementation are limited. In healthy horses, decreases in heart rate and changes in serum electrolyte concentrations have been reported.

**Hypothesis:**

Calcium gluconate administration at a rate of 0.4 mg/kg/min to eliminated endurance horses with metabolic problems will affect heart rate, gastrointestinal sounds, and serum electrolyte concentrations.

**Animals:**

Endurance horses eliminated from the Tevis Cup 100‐mile (160 km) endurance ride for metabolic problems and requiring IV fluid therapy were eligible.

**Methods:**

Sixteen horses were randomly assigned to receive 0.4 mg/kg/min of calcium (23% calcium gluconate solution) over 1 hour diluted in 10 L of a non‐calcium containing isotonic crystalloid (CAL group) or 10 L of a non‐calcium containing isotonic crystalloid (CON group). Staff members administering the fluids were blinded to treatment group. Blood samples were collected and physical examinations performed before and after treatment. Heart rates were recorded every 15 min during fluid administration. Data were compared using 2‐way analysis of variance (ANOVA) with repeated measures for continuous variables and Fisher's exact test for categorical variables.

**Results:**

Calcium was associated with lower heart rates 45 min after starting the infusion (*P* = .002). Gastrointestinal sounds were less likely to improve in the calcium group compared with the control group (*P* = .005). An increase in plasma phosphorus concentration (*P* = .03) was associated with calcium administration.

**Conclusions:**

Intravenous calcium supplementation to endurance horses eliminated from competition after development of metabolic problems may decrease heart rate but impairs improvement in gastrointestinal sounds.

**Abbreviations**
CK: creatine kinase
AST: aspartate aminotransferase
ANOVA: analysis of variance

## 1 INTRODUCTION

Fluid resuscitation is an important component of equine emergency medicine. Numerous options are available for the type of resuscitation fluid; dextrose, calcium, and potassium are commonly administered concurrently. The supplementation of resuscitation fluids with 23% calcium gluconate is used for a variety of clinical conditions including synchronous diaphragmatic flutter, weakness (when hypocalcemia is suspected), and life‐threatening hyperkalemia.^1^, ^2^, ^3^ Calcium supplementation also is commonly used in the resuscitation fluid for exercising horses treated in the field with metabolic problems, and particularly when laboratory testing is not available.^4^ This practice is based on previous observational research and documentation of hypocalcemia in endurance horses with metabolic problems.^5^

The research documenting the effects of calcium gluconate administration to horses has been completed primarily in healthy animals.^6^, ^7^, ^8^, ^9^ Some studies showed a decrease in heart rate during and after calcium administration, but rates remained within the normal range. Observed changes in serum electrolyte concentrations include increases in phosphorus and decreases in magnesium and potassium.^7^ One study evaluated calcium administration in clinical cases with gastrointestinal disease, but the study was not blinded or randomized.^10^ Based on current research in horses, it is difficult to know whether calcium supplementation in treatment fluids is warranted for clinical cases, particularly when testing of serum calcium concentrations and documentation of hypocalcemia may not be available. Research is needed to determine if the effects seen in healthy horses also will be present in clinically compromised horses that may or may not have hypocalcemia.

Our purpose was to prospectively evaluate the effects of calcium gluconate supplementation on the resuscitation of endurance horses requiring emergency care regardless of their calcium status. The hypothesis being tested was that calcium supplementation in the initial fluids will have clinical effects including decreased heart rate, improved gastrointestinal sounds, and a decreased need for additional IV fluids. Additionally, we hypothesized that calcium supplementation would be associated with biochemical changes including increases in plasma ionized and total calcium concentrations, as well as increases in plasma phosphorus concentration.

## 2 MATERIALS AND METHODS

The study took place at the Tevis Cup 100‐mile (160 km) endurance ride. The study was approved by the Institutional Animal Care and Use Committee of the UC Davis School of Veterinary Medicine and client consent was obtained for each animal.

Horses were treated at centers located at the 40, 58, 111, or 160 km mark on the trail depending on the location where they were eliminated. All horses disqualified for metabolic reasons as determined by a ride veterinarian were eligible for entry into the study, as well as horses that were disqualified for other reasons but developed clinical signs requiring IV fluid therapy. These metabolic conditions included, but were not limited to, acute abdominal disease (colic), exertional myopathy, and poor cardiovascular recovery, and have been described previously in endurance horses.^11^ Horses were excluded from the study if they had been treated with IV fluids before admission to 1 of the designated study treatment centers or if the horses were exhibiting synchronous diaphragmatic flutter.

### 2.1 Study design

All personnel were blinded to the treatment group until after the study was completed. After admission to a treatment center, each horse received a routine physical examination. Rectal temperature, heart rate, gastrointestinal sounds, and capillary refill time were recorded. Gastrointestinal sounds were recorded as absent, decreased, or normal and transformed to a numerical value of 0, 1, or 2, respectively, and reported as a categorical variable. Blood samples were collected from the jugular vein and placed into evacuated tubes containing potassium EDTA and sodium heparin. One of the tubes containing sodium heparin was centrifuged, and plasma was collected and transferred to storage at −80°C within 12 hours.

A 14‐gauge IV catheter was placed in a jugular vein and fluids were administered by gravity flow through an IV infusion set (STAT IV Set, International Win Ltd. Kennett Square, PA). Each horse was assigned a treatment code with a corresponding IV fluid. The personnel administering the fluid and the treatment veterinarian were blinded to the treatment group. Treatment codes were assigned according to a block randomization design based on treatment centers described above. Horses in the control group (CON) received a 10 L bolus of a commercially available acetated fluid (Plasmalyte A, Baxter, Deerfield, IL) over approximately 1 hour. Horses in the calcium group (CAL) received a 10 L bolus of a commercially available acetated fluid (Plasmalyte A, Baxter, Deerfield, IL) that contained 500 mL of 23% calcium gluconate (VetOne, Boise, ID) over approximately 1 hour. For a 450 kg horse, this rate of administration is approximately 0.4 mg/kg/min of calcium. After the initial 10 L fluid bolus, additional fluids were given at the discretion of the attending veterinarian.

During fluid bolus administration, heart rates were recorded at 15, 30, and 45 min after the start of the bolus. If any horse's heart rate decreased below 30 beats per minute (bpm), the study protocol called for the calcium administration to be stopped and the horse removed from the study. At the end of the 10 L bolus of fluids in both groups (1 hour), physical examinations were repeated and blood samples collected. The study was terminated when the 10 L bolus had been administered and the final examination had been completed and a blood sample had been collected. Horses were allowed free access to water during the study, but the amount of fluids consumed orally was not recorded. Horses with colic initially were restricted from feed, but all other horses had free access to grass hay and alfalfa hay. Oral electrolyte supplementation was not permitted during the study, but administration by owners before elimination from the race was not recorded.

### 2.2 Sample analysis

Plasma electrolyte, lactate, and creatinine concentrations were measured within 1 hour of collection using a commercial point‐of‐care analyzer (EPOC, Heska Analyzers, Loveland, CO). Biochemistry profiles were analyzed on plasma within 12 hours after collection using a commercial chemistry analyzer (Element DC5, Heska Analyzers, Loveland, CO). The saved samples were used to measure total plasma calcium concentration, as well as CK and AST activities and were analyzed within 30 days of collection using an additional commercial chemistry analyzer. These 3 tests were completed on a separate machine because of the presence of high test results that required dilution or were above the upper limit of quantification of the first analyzer.

### 2.3 Statistical analysis

Data are reported as mean (±SD) or median (range) depending on whether the data was normally distributed or not. Data were evaluated for normality using the Komolgorov‐Smirnov test. Admission data (pre‐treatment) for continuous variables were evaluated for effective randomization between groups using an unpaired *t* test or Mann‐Whitney test depending on whether they were normally distributed or not, respectively. Admission data (pre‐treatment) for categorical variables were evaluated for effective randomization using Fisher's exact test. Clinical and clinicopathological data between treatment groups were compared over time using 2‐way analysis of variance (ANOVA) with repeated measures except for gastrointestinal sounds which were compared between treatment groups for improvement using Fisher's exact test. The amount of additional (post‐study) fluids was compared between control and treatment groups using Fisher's exact test. A commercially available statistical software program (GraphPad Prism version 8.2.1 for Windows, GraphPad Software, La Jolla, CA) was used and a significance level of *P* < .05 was used.

## 3 RESULTS

Sixteen horses were enrolled in the study. Nine horses were in the control group and 7 were in the treatment (calcium) group. Baseline characteristics of the 16 horses are included in Table 1. All horses enrolled in the study recovered completely with treatment and none of the horses required referral to an equine hospital. Only 1 horse showed clinically relevant signs of colic.

**TABLE 1:** Baseline characteristics of the 2 study groups.

Variable	Control Group (CON)	Treatment Group (CAL)	*P* value
Sample size	9 horses	7 horses	N/A
Age (years)	14 ± 3	11 ± 4	.17
Breed	9 Arabians	5 Arabians	.18
		1 Arabian Cross	
		1 Mustang	
Sex	5 geldings	4 geldings	.99
	4 mares	3 mares	
Diagnosis at time of admission	5 Poor Recovery	6 Poor Recovery	N/A
	1 Colic	1 Dehydration	
	1 Dehydration		
	1 Musculoskeletal		
Distance at time of treatment (km)	1 horse at 40 km	1 horse at 40 km	.70
	3 horses at 58 km	3 horses at 58 km	
	5 horses at 111 km	3 horses at 111 km	

Randomization was successful for all variables except for bicarbonate. The control group had a bicarbonate concentration at T0 of 23.8 ± 1.3 mEq/L, which was significantly lower than the calcium group bicarbonate concentration at admission of 26.3 ± 1.8 mEq/L (*P* < .01). No significant interaction effect for this variable was found using 2‐way ANOVA.

**FIGURE 1:**
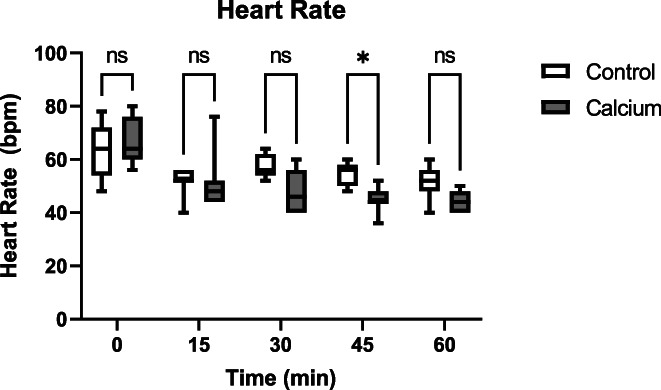
Heart rate during treatment with either isotonic crystalloid fluids containing calcium or isotonic crystalloid fluids containing no calcium. ns, not significant; *, significant difference between groups.

**FIGURE 2:**
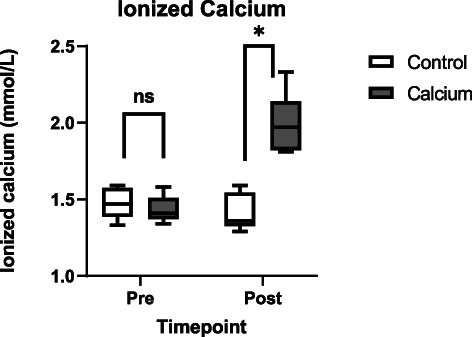
Ionized calcium concentration before and after treatment with polyionic fluids with or without supplementation with calcium gluconate.

Data for all groups (Control group‐ before IV fluids, Control Group—after IV fluids, Calcium group—before IV fluids, Calcium group—after IV fluids) are shown in Table 2. For gastrointestinal sounds, in the control group before treatment, 4 horses had a score of 0, 3 horses had a score of 1, and 2 horses had a score of 2. In the calcium group before treatment, 4 horses had a score of 1 and 3 horses had a score of 2. In the control group after treatment, 1 horse had a score of 1 and 8 horses had a score of 2. In the calcium group after treatment, 3 horses had a score of 1 and 4 horses had a score of 2. For the calcium group, only 2/7 (29%) required additional IV fluids before being released to the owners. The difference was not significant (*P* = .36). Two‐way ANOVA with repeated measures identified significant treatment‐time interactions with a decrease in heart rate associated with calcium treatment at the 45 min timepoint (Figure 1). Other variables showing significant treatment‐time interactions included plasma phosphorus concentration, CK and AST activities, plasma ionized (Figure 2) and total calcium concentrations. Specifically, calcium administration was associated with less improvement in gastrointestinal sounds (*P* = .03), increased plasma phosphorus concentrations (*P* = .03), decreased CK and AST activities (*P* = .046), and increased plasma ionized and total calcium concentrations (*P* < .0001). Improvement in gastrointestinal sounds occurred in only 2/7 (29%) of horses in the calcium group but in 9/9 (100%) of horses in the control group (*P* < .01).

**TABLE 2:** Clinical and biochemical variables for the CON (control) and CAL (calcium) groups before and after treatment.

Variable	Time 0 (Control)	Time 60 (Control)	Time 0 (Calcium)	Time 60 (Calcium)	*P* value (overall, control, calcium)
Temperature (C)	37.8 ± 0.3	37.5 ± 0.6	37.8 ± 0.4	37.7 ± 0.5	.9
Heart rate (bpm)	63 ± 11	52 ± 6	68 ± 9	45 ± 4	.002, .9, .1
Capillary refill time (s)	2.1 ± 0.6	1.7 ± 0.4	2.0 ± 0.8	1.6 ± 0.5	.6
BUN (mg/dL)	34.8 ± 9.5	35.7 ± 7.0	30.5 ± 4.6	28.3 ± 3.6	.16
Creatinine (mg/dL)	1.3 ± 0.5	1.3 ± 0.3	1.3 ± 0.3	1.2 (0.9‐1.2)	.21
Phosphorus (mg/dL)	4.5 ± 1.5	4.7 ± 1.6	3.7 ± 0.9	5.9 ± 1.5	.03, .94, <.01
Total protein (mg/dL)	7.9 ± 0.7	6.4 ± 2.3	7.7 ± 0.5	6.0 ± 0.3	.27
Albumin (mg/dL)	3.7 ± 0.4	3.0 ± 1.1	3.7 ± 0.3	2.9 ± 0.3	.38
Globulin (mg/dL)	4.2 ± 0.6	3.4 ± 0.4	4.0 ± 0.4	3.1 (2.4‐3.5)	.52
AST (IU/L)	468 (325‐1076)	383 (278‐996)	4852 ± 6651	4327 ± 5619	.05, .96, .02
CK (IU/L)	1661 ± 1442	1163 (458‐4107)	123 744 ± 189 669	20 915 (418‐404 569)	.05, .99, .02
GGT (IU/L)	31 ± 13	22 (15‐58)	26 ± 6	18 ± 6	.57
Total Bilirubin (mg/dL)	3.0 ± 1.2	1.7 (1.4‐3.9)	2.7 ± 1.0	2.2 ± 0.8	.40
Sodium (mEq/L)	136 ± 6	135 ± 5	135 ± 4	133 ± 3	.15
Potassium (mEq/L)	3.2 (2.4‐4.9)	3.1 (2.3‐4.8)	2.8 ± 0.5	2.9 ± 0.5	.13
Chloride (mEq/L)	99 ± 7	99 ± 5	97 ± 6	94 ± 3	.24
Ionized Ca (mmol/L)	1.5 ± 0.1	1.4 ± 0.1	1.4 ± 0.1	2.0 ± 0.2	<.0001, .25, <.0001
Glucose (mg/dL)	140 ± 39	136 ± 31	131 ± 41	119 ± 27	.44
Hct (%)	44 ± 7	34 ± 7	45 ± 3	34 ± 2	.40
Lactate (mmol/L)	1.5 (1.0‐4.1)	1.3 ± 0.3	2.2 ± 0.8	1.0 ± 0.2	.08
Bicarbonate (mEq/L)	24.3 (21.3‐25.3)	23.1 ± 1.5	26 ± 1.8	25.4 ± 1.7	.69
Total Calcium (mg/dL)	12.1 (11.3‐14.1)^k^	11.3 ± 0.8^k^	12.7 ± 1.4^l^	17.5 ± 1.7^l^	<.0001, .15, <.0001

*Note*: First *P* value represent results of the 2‐way ANOVA for the interaction between treatment (calcium and control) and change between the time 0 and time 60 min points. If interaction was statistically significant, second *P* value represents comparison for control group (pre vs. post treatment) and third *P* value represents comparison for calcium group (pre vs. post treatment). Data are reported as mean ± SD for normally distributed data and median (range) for non‐normally distributed data.

After the end of the study, 5/9 (56%) horses in the control group required additional IV fluids before being released to the owners.

## 4 DISCUSSION

Intravenous infusion of 23% calcium gluconate at a rate of approximately 0.4 mg/kg/min of calcium diluted in 10 L of isotonic crystalloid fluids over approximately 1 hour resulted in increased plasma calcium concentrations. At approximately 45 min after the start of infusion, a decrease of 10 bpm in heart rate would be expected as compared with horses that received IV fluids that do not contain calcium. Small increases in plasma phosphorus concentration are apparent when this amount of calcium gluconate is added to the IV fluids to be administered. Interestingly, gastrointestinal sounds do not appear to improve as much when calcium gluconate is added to IV fluids at the rate used in our study.

A decrease in heart rate with calcium administration previously has been reported in horses and other species.^6^, ^12^, ^13^, ^14^ In another study in horses using a similar dosage and rate of calcium administration as in our study, heart rates decreased from baseline by approximately 5 bpm in healthy horses.^6^ However, in our study, heart rates decreased from baseline by approximately 20 bpm and decreased compared with the CON (no calcium) group by approximately 10 bpm. This more marked decrease in heart rate as compared with previous reports may be a consequence of the higher baseline heart rates at the beginning of infusion given that the horses in this study were clinical cases as opposed to healthy animals. Additionally, the prior study did not indicate that crystalloid fluids were administered simultaneously and therefore the larger decrease in heart rate also could have been associated with the addition of 10 L of IV fluids used to treat hypovolemia.^6^

Hypercalcemia has been associated with bradycardia in numerous case reports or smaller studies. However, there is not widespread agreement on the mechanism.^12^, ^13^, ^14^ Previous reports in horses have shown an improvement in cardiac index (despite decreases in heart rate), which could have resulted in improved perfusion with subsequent improvement in heart rate in our study.^6^ Although not significant, lactate concentrations improved with calcium administration (*P* = .08), which might be consistent with an improvement in perfusion. Improvements in cardiac index and perfusion theoretically could decrease the need for additional IV fluids during treatment.

Another possible mechanism for the development of bradycardia after calcium administration could be related to calcium‐mediated vasoconstriction inducing hypertension and reflex bradycardia. Vasoconstriction associated with calcium infusion has been described but was not present in all studies.^12^, ^15^ Future studies could evaluate changes in blood pressure associated with calcium administration to clinical horses.

Hypercalcemia also may cause bradycardia by its direct effects on the heart. Specific effects of calcium infusion on the myocardium include an increase in the threshold potential, a shift of the curve relating V_max_ to the resting potential, and an increase in the magnitude of the calcium inward current.^16^ To our knowledge, the exact mechanisms by which these effects create bradycardia has not been completely elucidated.

Plasma total calcium concentrations at the end of this study in the CAL group were 17.5 ± 1.7 mg/dL, which is similar to the concentrations observed in a previous study that used an infusion rate of 0.4 mg/kg/min.^6^ Another study reached serum total calcium concentrations of 21.4 ± 1 mg/dL using a slower infusion rate over a longer period of time (2 hours as opposed to 1 hour in the present study). These other studies were performed in healthy horses, and this difference may explain the small variations in the observed total calcium concentrations among studies. These high calcium concentrations are supraphysiological and require further study with regard to potential additional effects on cardiovascular physiology and possible undetected adverse effects.

Creatine kinase and aspartate aminotransferase activities were decreased in association with calcium administration in our study. This finding was unexpected, but could be related to differences between the control and treatment groups. In 2 small studies in cows, a decrease in these enzyme activities was not observed with calcium administration.^17^, ^18^ To our knowledge, the effects of calcium gluconate infusion on muscle enzyme activities have not been reported. Hypercalcemia has been reported in association with rhabdomyolysis in people, but in our study no relationship was found between CK activity before infusion and plasma ionized calcium concentration.^19^

Although randomization of the 2 groups in our study was technically successful (CK and AST activities were not significantly different before the start of the infusions), 2 horses in the calcium group had CK activities >100 000 IU/L. In contrast, no horses in the control group had CK activities >100 000 IU/L. However, neither of the horses was admitted to a treatment center at the start of the study with a presenting complaint of rhabdomyolysis. Instead, both horses presented for poor heart rate recovery, and tight muscles were palpated during treatment administration. The severity of the rhabdomyolysis was not identified until the plasma biochemistry panels were performed. Given the subjective imbalance between the groups in our study, additional research is warranted to investigate the effects of calcium on horses with rhabdomyolysis because of the potential bias these 2 horses with markedly increased enzyme activities pose to the small group of treatment horses. However, in this small number of horses, calcium administration did not appear to have any detectable negative effects on endurance horses with rhabdomyolysis.

Another unexpected finding in our study was that gastrointestinal sounds improved more in the control group than in the group that received calcium. A priori, we had hypothesized that calcium supplementation would improve gastrointestinal function because of the potential effects of calcium on gastrointestinal motility. Interestingly, administration of calcium to nomocalcemic cattle also had negative effects on gastrointestinal function.^20^ Further research is needed, but this finding may represent a potential reason to avoid calcium supplementation in the resuscitation fluids of normocalcemic horses, especially at the higher doses used in our study. Calcium plays a role in the vasoconstriction of splanchnic vessels, which might lead to decreased gastrointestinal sounds.^21^

The rate of calcium administration in our study (0.4 mg/kg/min) was the same or higher than the rate used in previous studies, which ranged from 0.1 to 0.4 mg/kg/min.^6^, ^7^ This rate was chosen because of its similarity to previous studies and because we use a similar rate in the treatment of clinically affected endurance horses. In 1 horse in our study, plasma ionized calcium concentrations reached 2.33 mmol/L and plasma total calcium concentrations reached 19.8 mg/dL. The concern for bradycardia is a primary reason for avoiding high rates of calcium administration and high concentrations of calcium, but the rate of 0.4 mg/kg/min used in our study was not associated with bradycardia. Increases in contractility also have been described with calcium administration, but this effect was not evaluated in our study.^6^

In general, horses in our study were not hypocalcemic. Decreased calcium concentrations have been observed in endurance horses during competition, but development of hypocalcemia is variable.^5^, ^22^ If the study group horses had been more hypocalcemic, it may have been possible to observe more improvement in clinical and biochemical variables. Similarly, the horses in our study may only have had mild to moderate clinical exhaustion based on the clinical and laboratory findings. If more severely exhausted horses had been included, the effects of calcium supplementation could have been more substantial.

Plasma phosphorus concentration increased in the calcium group of horses in our study, which is similar to results of a previous study that found serum phosphorus concentration increased from 3.4 ± 0.3 mg/dL at baseline to 4.8 ± 0.7 mg/dL after treatment.^7^ In the previous study, the final calcium infusion rate was approximately 0.25 mg/kg/min. However, lower rates were used at the beginning of the infusion.^7^ In our study, a rate of 0.4 mg/kg/min was used and plasma phosphorus concentration significantly increased from 3.7 ± 0.9 mg/dL at baseline to 5.9 ± 1.5 mg/dL at the end of the 60‐min infusion. Clinical effects from this presumably transient hyperphosphatemia would not be expected, but this possibility remains to be studied.

One of the important limitations of our study was the smaller than expected number of horses eligible for enrollment. Based on a power calculation performed a priori using an alpha of 0.05 and power of 0.80, a total of 24 enrolled horses was expected to be needed to detect significant differences in heart rate. A mean heart rate of 44 bpm was used based on reported values from previous studies treating endurance horses.^4^ Despite the small sample size, significant effects on heart rate were observed, but changes in other variables such as lactate might have been significant with a larger sample size.

Another limitation of our study was that a single dose of calcium gluconate was used. If multiple doses (especially lower doses) and a larger number of horses had been used, it may have been possible to determine a rate of calcium infusion at which some of the effects observed in the study did not develop.

In conclusion, our results suggest that 0.4 mg/kg/min of calcium in the form of 23% calcium gluconate can be infused IV to endurance horses requiring emergency care as an addition to 10 L of isotonic crystalloids. However, supraphysiological concentrations of calcium are achieved, as well as increases in plasma phosphorus concentrations, the effects of which require further study. Heart rates would be expected to decrease by approximately 10 bpm. Improvements in muscle enzyme activities and negative effects on gastrointestinal sounds require further research. All of these findings may be dose dependent.

## CONFLICT OF INTEREST

The authors declare no conflict of interest.

## OFF‐LABEL ANTIMICROBIAL DECLARATION

Authors declare no off‐label use of antimicrobials.

## INSTITUTIONAL ANIMAL CARE AND USE COMMITTEE (IACUC) OR OTHER APPROVAL DECLARATION

Approved by the University of California, Davis IACUC, Protocol 22 372.

## HUMAN ETHICS APPROVAL DECLARATION

Authors declare human ethics approval was not needed for this study.
